# Interaction of cochlin and mechanosensitive channel TREK-1 in trabecular meshwork cells influences the regulation of intraocular pressure

**DOI:** 10.1038/s41598-017-00430-2

**Published:** 2017-03-28

**Authors:** Teresia A. Carreon, Aida Castellanos, Xavier Gasull, Sanjoy K. Bhattacharya

**Affiliations:** 1 Bascom Palmer Eye Institute, University of Miami, Miami, Florida, USA; 2 Department of Biochemistry and Molecular Biology, University of Miami, Miami, USA; 3 Department of Biomedicine, University of Barcelona, Barcelona, Spain; 4 Institut d’Investigaciones Biomediques August Pi I Sunyer (IDIBAPS), Barcelona, Spain; 5 Institute of Neurosciences, University of Barcelona, Barcelona, Spain

## Abstract

In the eye, intraocular pressure (IOP) is tightly regulated and its persistent increase leads to ocular hypertension and glaucoma. We have previously shown that trabecular meshwork (TM) cells might detect aqueous humor fluid shear stress via interaction of the extracellular matrix (ECM) protein cochlin with the cell surface bound and stretch-activated channel TREK-1. We provide evidence here that interaction between both proteins are involved in IOP regulation. Silencing of TREK-1 in mice prevents the previously demonstrated cochlin-overexpression mediated increase in IOP. Biochemical and electrophysiological experiments demonstrate that high shear stress-induced multimeric cochlin produces a qualitatively different interaction with TREK-1 compared to monomeric cochlin. Physiological concentrations of multimeric but not monomeric cochlin reduce TREK-1 current. Results presented here indicate that the interaction of TREK-1 and cochlin play an important role for maintaining IOP homeostasis.

## Introduction

Glaucoma refers to a group of irreversible blinding diseases. Primary open angle glaucoma (POAG) is a disease of exclusion, when no illness or injury can be ascribed to glaucoma. In all forms of glaucoma the only proven protection strategy to delay the progression is reduction in intraocular pressure (IOP). In a significant number of glaucoma and in all POAG patients glaucoma is associated with elevated IOP. Elevated IOP occurs due to impeded outflow of aqueous humor, the clear fluid that bathes the anterior eye chamber. Increased resistance that develops in the trabecular meshwork (TM), a filter-like region in the anterior chamber, is attributed to increased IOP. Our unbiased proteomic analyses have consistently detected the presence of the extracellular matrix (ECM) protein cochlin in pathologic TM but not in control^1^. We also found the presence of cochlin in DBA/2J mice^2^, which develops spontaneous IOP elevation^3, 4^. Our comprehensive analyses with a double Gonio lens microscope, optical coherence tomography (OCT), and slit lamp identified a subset of DBA/2J mice where pigmentary dispersion is negligible and the angle remains open; yet, IOP remains elevated for nearly a month. The endpoint Fontana-Masson staining confirmed a lack of pigmentary dispersion^5^ in a subset of DBA/2J mice which shows correlation of elevated cochlin expression with high IOP, which is corroborated by non-invasive antibody mediated OCT analyses^6^. Previously, our investigation revealed syn-expression of TREK-1 upon induction of cochlin expression^7^. Our previous cell biology experiments have suggested syn-expression of cochlin and the stretch-activated channel TREK-1^7^. Cochlin possesses von Willebrand factor A (vWFA) like domains, which, like vWFA domains are capable of multimerization^1^. Cochlin plays a role in TM filopodia formation and other aspects of cell shape and behavior. The mechanosensitive K^+^ channel TREK-1 is typically activated by shear stress, membrane stretch, and cell swelling^8–12^. In other fluid flow regimes, TREK-1 alone is capable of detecting shear stress changes to elicit a response in order to modulate its electrical activity^13–16^. Whether syn-expression of cochlin and TREK-1 leads to their interaction with TREK-1 in order to effectively regulate IOP remains unknown. The magnitude of vWFA multimerization within physiological range is governed by the degree of shear stress^17^. The degree of vWFA multimerization results from the interaction of different protein interactors resulting in different biological consequences. Information on differences between the interactions of multimeric versus monomeric cochlin is completely lacking. Although regulation of cochlin has been shown to be important for IOP and elevated cochlin results in elevated IOP^18^, whether cochlin and TREK-1 are needed together to generate broad IOP changes has not been investigated. Here we present evidence that cochlin and TREK-1 are both required components for IOP regulation. We present evidence that pathologic state associated multimeric cochlin alters biochemical and functional electrophysiological properties differently than monomeric cochlin.

## Results

### Cochlin and TREK-1 are necessary components for IOP regulation

**Figure 1:**
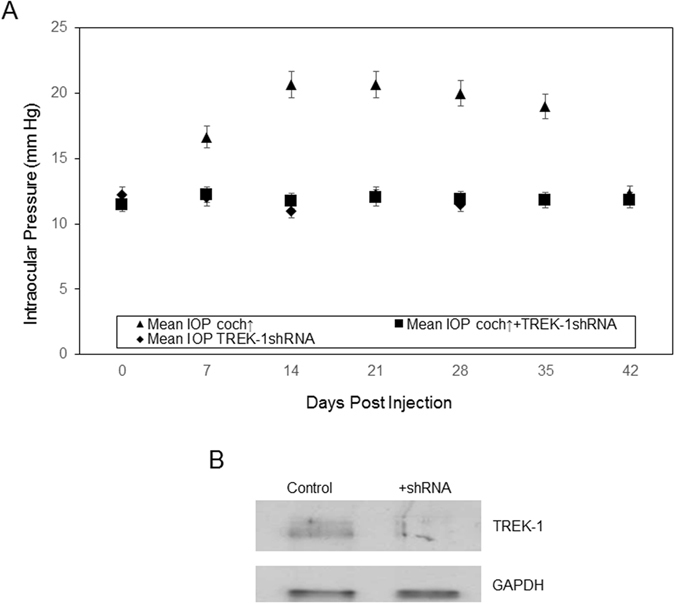
TREK-1 silencing inhibits cochlin overexpression increase in IOP. (**A**) Injection of cochlin-expression lentivirus (▲), cochlin-expressing lentivirus with TREK-1 shRNA (■), or TREK-1 shRNA alone (♦) into DBA/2J-Gpnmb+/SjJ mice TM. Analyses of variance (ANOVA) showed a statistically significant difference between the three groups. Scheffe’s post hoc test showed that cochlin alone produced an increase in IOP that was statistically different from the maintenance of a lower IOP in cochlin + TREK-1 shRNA (n = 6; *p < 0.016) and TREK-1 shRNA only (n = 6; *p < 0.016) treated groups. (**B**) A representative Western blot of control and TREK-1 shRNA treated mice (DBA/2J-Gpnmb+/SjJ) TM as indicated. The blot was sequentially probed (with a stripping step prior to second probing) with antibodies to TREK-1 and GAPDH as indicated.

The DBA/2J-Gpnmb+/SjJ mouse is a genetically matched strain to that of the DBA/2J mouse however, DBA/2J-Gpnmb+/SjJ mice do not develop elevated IOP, nor do they demonstrate glaucomatous optic nerve damage^19^. We have previously demonstrated an increase in IOP due to over expression of cochlin in DBA/2J-Gpnmb+/SjJ mice^18^. TREK-1 was silenced in DBA/2J-Gpnmb+/SjJ mouse TM through the use of shRNA (Fig. 1). In this particular experiment, channel silencing prevented the cochlin-induced increase in IOP when compared to control animals that were injected with cochlin alone (n = 6; p < 0.016), demonstrating that both proteins are needed for IOP regulation. TREK-1 silencing without cochlin overexpression maintains a lower IOP similar to what is observed with cochlin overexpression and TREK-1shRNA in combination (n = 6; p < 0.016) (Fig. 1A). The TREK-1shRNA reduced TREK-1 expression by ~70% (Fig. 1B) compared to control samples. Following the establishment of these components in IOP regulation, we asked whether interaction between them elicits spatial changes in the TM by altering the extracellular matrix of TM cells^7, 20^.

### Interaction of both proteins produce changes in TM cell architecture towards homeostatic regulation of fluid flow

**Figure 2:**
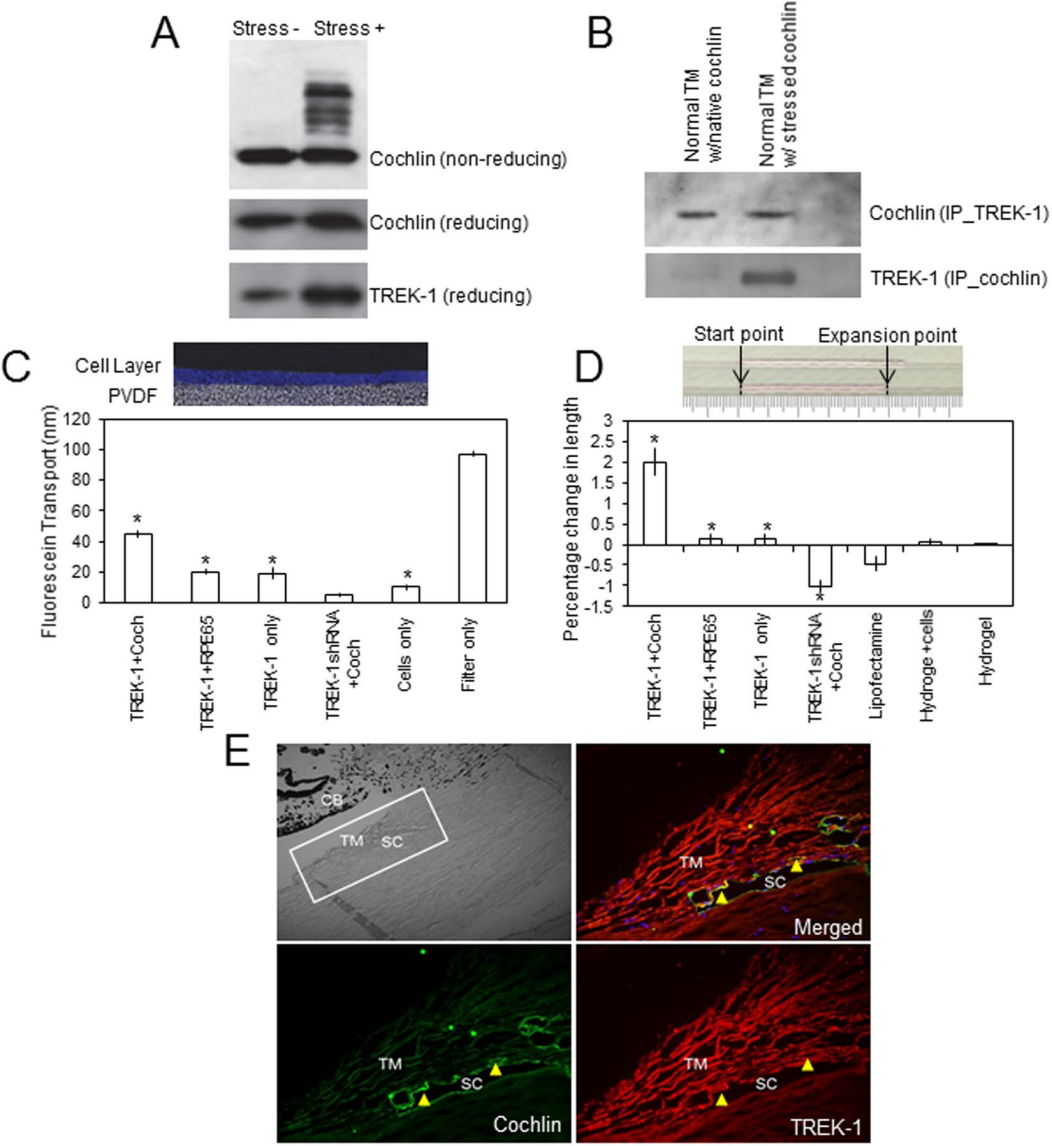
Interaction between cochlin and TREK-1 results in changes in fluid flow dynamics. (**A**) Cochlin multimerization under shear stress increases the amount of TREK-1 pulled down as shown through western blot analysis. Upper panel shows cochlin with or without shear stress as indicated on a western blot after separation on a non-reducing but denaturing gel, middle and lower panels are identical experiments as above but from reducing and denaturing gels probed with cochlin and TREK-1 antibodies as indicated. (**B**) Co-immunoprecipitation followed by western blot analysis details the interaction of cochlin and TREK-1 in normal as well as glaucomatous samples (the latter is presumably in the presence of shear stress). (**C**) Fluorescein dye migration was measured as fluorescence intensity across several layers of cells on a PVDF membrane within an Ussing-type chamber (as shown in top figure) in the presence of TREK-1 + cochlin, TREK-1 + RPE65, TREK-1 only, TREK-1 shRNA + cochlin as indicated. Student’s t-test showed significant difference in fluorescein transport between TREK-1 + cochlin and all other transfected groups (n = 10; *p < 0.001). TREK-1 + cochlin also showed significant difference in dye transport with non-transfected cells (n = 10; *p < 0.001). (**D**) TM cells transfected with TREK-1 + cochlin, TREK-1 + RPE65, TREK-1 only, TREK-1 shRNA + cochlin, or untransfected (hydrogel + cells) as indicated were cultured with rat tail collagen and placed into capillary tubes. The amount of collagen expansion was measured and recorded. Expansion shown by TREK-1 + cochlin, TREK-1 + RPE65, and TREK-1 only were found significantly different than cells only (hydrogel + cells) by pairwise t-test. TREK-1 shRNA + cochlin showed a contraction, which was again statistically significant than cells only (n = 10 per group). (**E**) Immunohistochemistry of human TM sections probed for cochlin (red) and TREK-1 (green). The brightfield reference image indicates the locations of the trabecular meshwork (TM), Schlemm’s canal (SC), and ciliary body (CB).

**Figure 3:**
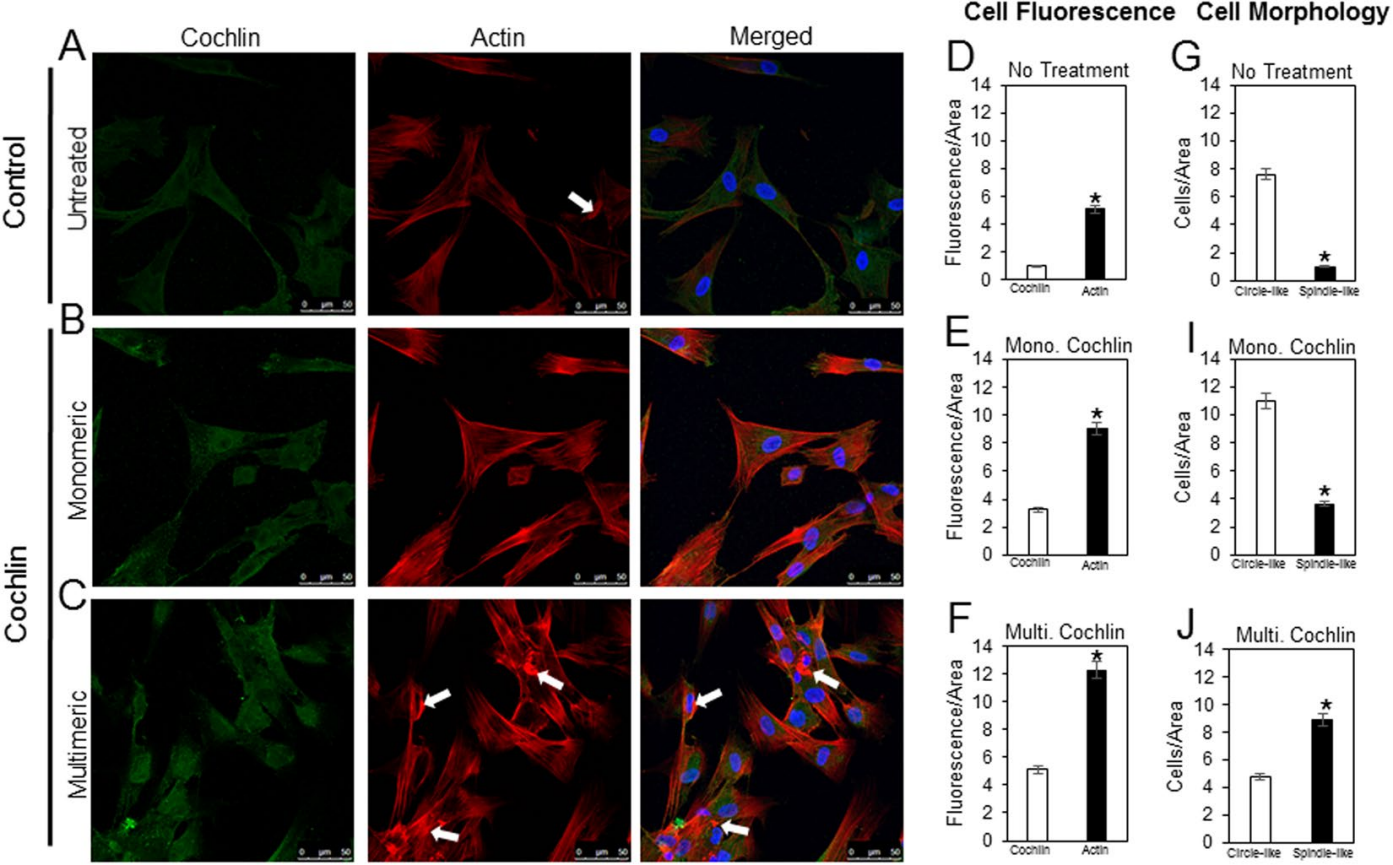
Molecular state of cochlin and cellular architecture of primary TM cells. (**A–C**) Representative immunocytochemical images of human normal TM cells probed for cochlin (green) and actin (red) after 24 hour incubation with exogenous monomeric or multimeric cochlin as indicated. White arrows are showing areas of actin clustering. (**D–F**) Quantification of normal TM cells under untreated, monomeric or multimeric cochlin conditions using fluorescence intensity/area. The fluorescence intensity was measured as a relative arbitrary unit using ImageJ software under the same settings and conditions for each sample. (**G–J**) Quantification of cellular morphology (circle-like or spindle-like) of untreated, monomeric, or mulltimeric cochlin treated cells. Cellular morphology has been depicted as a relative arbitrary unit of cells/area.

The interaction of cochlin and TREK-1 becomes an area of interest as both are components that seem to be needed for IOP regulation, as seen in the silencing experiments. Cochlin, that was multimerized through the application of shear stress, pulls down a greater amount of TREK-1 compared to monomeric cochlin in western blot analysis (Fig. 2A) performed with purified cochlin. This is consistent with the observation that similar amounts of cochlin are associated with greater amounts of TREK-1 in human TM samples, modeled through the application of shear stress (Fig. 2B). Cochlin in glaucomatous TM is likely in the multimeric form, perhaps either due to the presence of shear stress or oxidative stress. Shear stress causes an increase in cochlin thus resulting in an increase in TREK-1 interaction. To investigate if the cochlin-TREK-1 interaction produced significant rearrangements in cellular organization within the TM cells, we measured fluorescein dye transport using an Ussing-type chamber across a trilayer of TM cells cultured on a PVDF membrane (Fig. 2C). The TM cells were transfected with TREK-1+ cochlin (monomeric), TREK-1+ Retinal Pigment Epithelium-Specific Protein 65 kDa (RPE65), TREK-1 alone, TREK-1 shRNA, or were non-transfected controls. RPE65 was used as a control protein in this experiment because of its molecular similarity in size to cochlin. The filter alone allowed substantial flow of the dye while addition of the cells decreased this flow tremendously. TM cells transfected with TREK-1+ cochlin show a significant increase in fluorescein dye transport compared to the other transfected (TREK-1 only or TREK-1+ RPE65 or TREK-1 shRNA + cochlin; n = 10; *p < 0.001) or non-transfected cells (n = 10; *p < 0.001). The much higher magnitude of transport when cells are transfected using both TREK-1 + cochlin [compared to that with TREK-1 shRNA + cochlin (n = 10; *p < 0.001)] suggests that interaction of both proteins produces significant changes in cell shape due to cytoskeletal remodeling. This implies that both elements (TREK-1 and cochlin) are needed to alter cell architecture towards homeostatic regulation of fluid flow. Collagen gel assays were performed in order to further investigate TM cellular architecture (Fig. 2D). Interestingly, TREK-1 + RPE65 transfected cells and TREK-1 only transfected cells performed similarly with a minimal expansion compared to TREK-1 + cochlin transfected cells, which showed a significant increase in gel expansion (compared with cells only; n = 10; *p < 0.001). Downregulation of TREK-1 expression with TREK-1 shRNA transfected cells caused contraction rather than expansion. The contraction was significant compared to the cell only control (n = 10; *p < 0.001) or with TREK-1 + cochlin (n = 10; *p < 0.001). Immunohistochemistry of human TM sections shows the co-localization of both TREK-1 and cochlin specifically in the Schlemm’s canal and TM region (Fig. 2E). The close proximity of these components is key to enabling a potential interaction with one another in the TM tissue milieu. Immunocytochemistry on human normal TM cells in the presence of exogenous cochlin demonstrates a difference in cell shape as well as actin expression in the cytoskeleton (Fig. 3).

In the untreated cells (Fig. 3A) and in the presence of monomeric cochlin (Fig. 3B), cytoskeleton architecture remains visibly organized whereas in the presence of multimeric cochlin, actin expression increases along with clustering of actin fibers (Fig. 3C). The clustering, if at all present in the untreated cells, is very limited in intensity compared to that visualized with the multimeric cochlin treatment. A relative quantification of cochlin and actin in treated as well as untreated cells has been presented in Fig. 3D,F using fluorescence/area. Furthermore, a difference in the shape of the cells is present between all three cohorts (untreated, mono- and multi-meric cochlin treated). The untreated cells and the monomeric treated cells (Fig. 3A,B) exhibited a uniformed flattened, circular shape when compared to multimeric treated cells (Fig. 3C), which assumes a splindle-like conformation. Quantification further validated cell shape changes due to addition of different forms of cochlin (Fig. 3G,I,J). These observations are consistent with and corroborates our previous studies^7, 18^, demonstrating similar changes in cell shape and interaction of multimerized cochlin with TREK-1 channels^7^.

**Figure 4:**
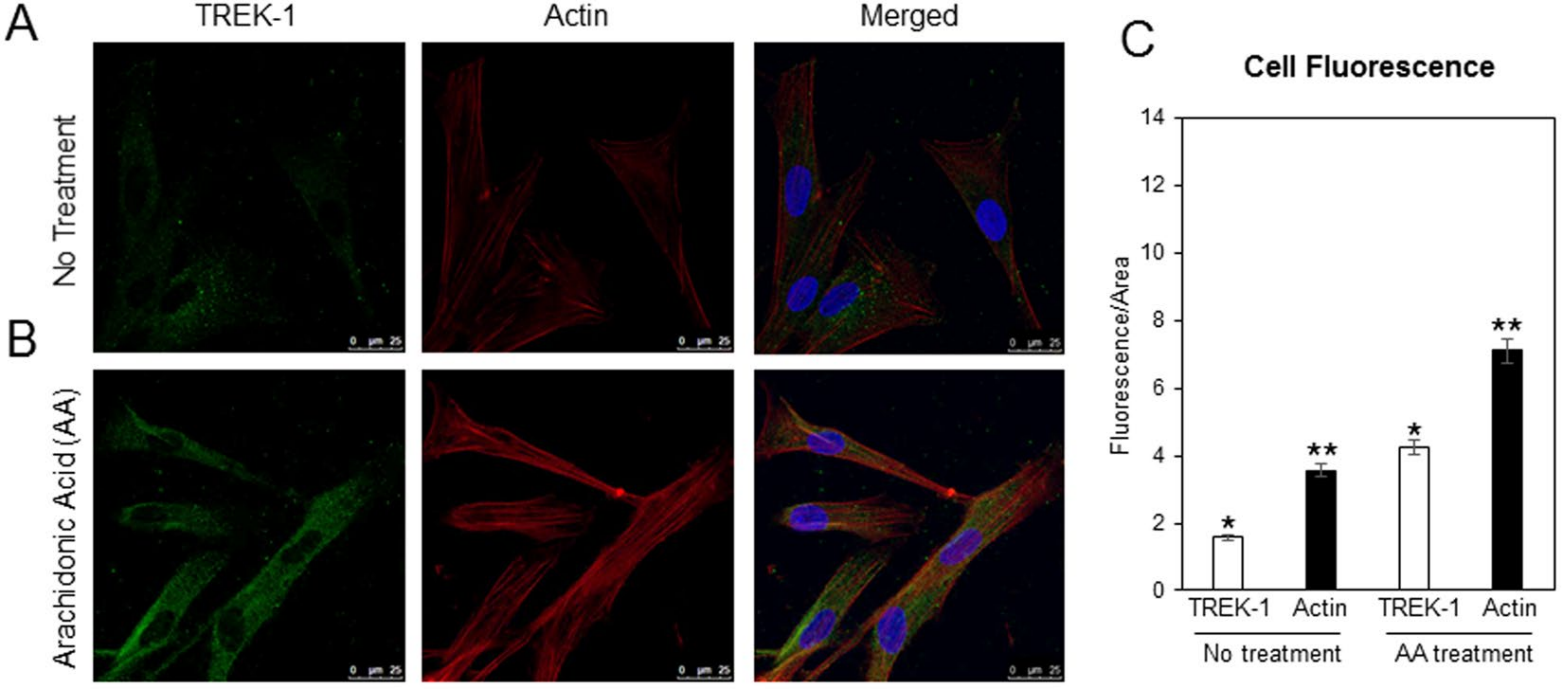
Effects of TREK-1 activation elicited by arachidonic acid (AA) on cellular architecture of primary TM cells. (**A** and **B**) Representative immunocytochemical images of human normal TM cells probed for TREK-1 (green) and actin (red) after no treatment or AA treatment. (**C**) Quantification of normal TM cells under no treatment or AA treatment conditions using fluorescence intensity/area. The fluorescence intensity was measured as a relative arbitrary unit using ImageJ software under the same settings and conditions for each sample.

To compare the changes in the actin cytoskeleton elicited by exogenous cochlin, further experiments were conducted to investigate the effects of TREK-1 channel activation. Previous observation has shown that cellular lipids such as arachidonic acid (AA)^21–23^ activate TREK-1 channels. We utilized AA in order to activate TREK-1 channels and observe changes in the actin cytoskeleton of TM cells (Fig. 4). TM cells were treated with 20 µM of AA and the actin cytoskeleton of the cells was analyzed using immunocytochemistry. In the untreated cells (Fig. 4A), TREK-1 expression remains at a normal state along with the actin. The AA treated cells (Fig. 4B) exhibit a robust increase in TREK-1 expression as well as actin. The increase of expression is further supported by the quantification in Figure C. These observations demonstrate the effects the activation of TREK-1 has on the actin cytoskeleton of TM cells. Inhibition of phospholipase A2, an enzyme that hydrolyzes AA, has been shown to cause a decrease in the actin cytoskeleton of porcine TM cells^21^, corroborating our results presented here. The observed change in cellular architecture is different from that found in the presence of exogenous multimeric cochlin (Fig. 3). Taken together with previous studies^7, 18^ these results suggest that the multimerized cochlin may affect the overall cellular architecture mediated by interaction with TREK-1. These results allude to the potential cytoskeletal changes taking place in the presence of multimeric cochlin that has resulted as a consequence of shear stress.

### Multimeric cochlin modulates TREK-1 channel current

**Figure 5:**
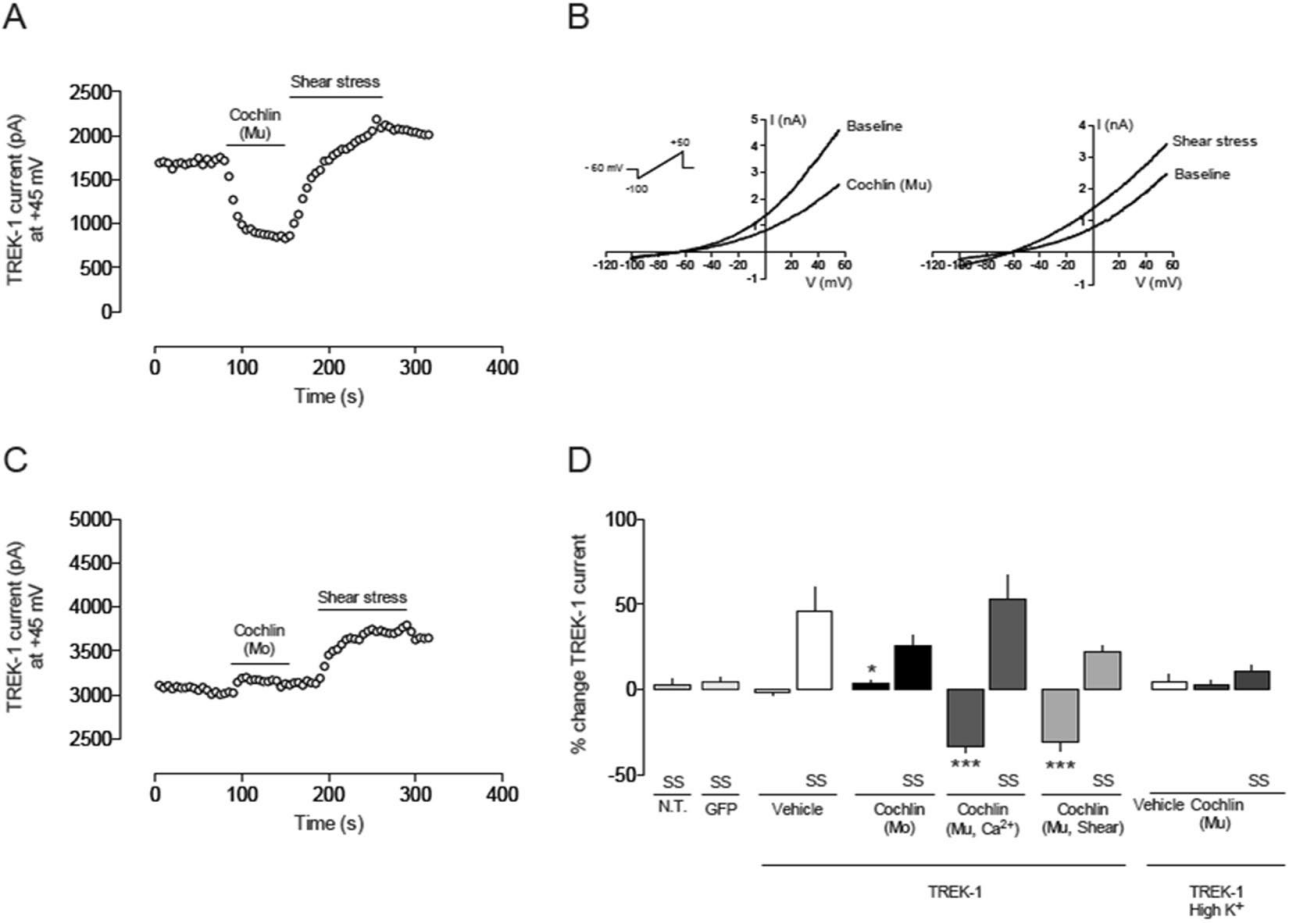
Whole-cell patch clamp recordings of TREK-1 in HEK293 cells (**A**) Time course of the effect of multimerized cochlin (10 nM) applied to the bath in a HEK293 cell expressing hTREK-1. Cochlin was previously multimerized by Ca^2+^ addition for 2 h. Using the whole-cell configuration of the patch clamp technique, TREK-1 current was activated with 1 s voltage ramps from −100 to + 50 mV every 5 s (as in **B**). Current values were measured at +45 mV in each ramp and plotted vs. time. Multimerized cochlin (Mu) produced a significant current reduction. In contrast, current increased when the cell was stimulated by shear stress (increase in bath perfusion rate). (**B**) Left: TREK-1 current elicited by a voltage ramp in basal conditions and after addition of multimerized cochlin. Right: an increase in bath perfusion rate activates TREK-1 channels and increases whole cell current. (**C**) Time course of the effect of monomeric (Mo) cochlin using the same protocol as in A. (**D**). Quantification of the effects of cochlin on hTREK-1 current in transiently transfected HEK293 cells: Vehicle (n = 11); monomeric cochlin (Mo; 10 nM; n = 8); monomeric); cochlin multimerized by Ca2+ addition for 2 h (Mu, 10 nM; n = 8) and cochlin multimerized by passing the cochlin solution (10 nM) through a 26 gauge syringe needle 20 times (n = 8). High K + solution: recordings of TREK-1 current in high extracellular K^+^ concentration (135 mM). Vehicle, multimeric cochlin (Mu; 10 nM) and shear stress stimuli were applied (n = 8). SS: shear stress. *p < 0.05; ***p < 0.01, Student’s t-test vs. vehicle.

To assess if cochlin interaction with TREK-1 also modulates the channel current, human TREK-1 was transiently expressed in HEK293 cells, and channel activity was monitored using whole-cell patch clamping. Cells expressing TREK-1 were voltage-clamped at −60 mV and voltage ramps were used to record the channel current. Multimerized cochlin, monomeric cochlin, or vehicle were applied to the bath (Fig. 5). TREK-1 basal channel activity was strongly reduced by multimerized cochlin at physiological concentrations (10 nM). Equivalent effects on the current were seen when cochlin multimerization was previously induced by addition of Ca^2+^ (−33.4 ± 4.1%; n = 8; Fig. 5A,B and D) or after a shear stress protocol aided by a syringe (−30.9 ± 5.1%; n = 8; Fig. 5D). The addition of vehicle did not produce significant effects (−1.6 ± 1.3%; n = 11). As previously reported^13–16^, fluid shear stress, induced by increasing the bath perfusion rate, produced an increase in TREK-1 activity (Fig. 5B). Despite the variability observed among cells in the stimulating effect of shear stress, no differences were seen between the shear stress effect on vehicle and cochlin groups (Fig. 5D). On the contrary, when monomeric cochlin was assayed, a very small but significant increase in TREK-1 activity could be observed compared with the vehicle (+3.8 ± 1.4%; n = 9; Fig. 5C and D).

It has been previously reported that external protons, heat, and pressure-evoked TREK-1 gating inputs act via a common gate that has the characteristics of a C-type gate^24^. For this, the extracellular region of the transmembrane segment M4 is a key element of the TREK-1 gating apparatus and despite whether the signal activates or inhibits the channel, the mechanism is conversed in different K_2_P channels. A mutation in the M4 segment (W275S) produces a gain of function TREK-1 channel with a reduced sensitivity to extracellular protons or temperature, an effect that can be mimicked by a high extracellular potassium concentration^24^. To test whether the inhibitory effect of multimeric cochlin on TREK-1 was mediated via the C-type gate, recordings on high K^+^ extracellular solution were performed. In contrast to the inhibitory effect of multimeric cochlin, no significant effect was observed when recording in high K^+^ solution (+2.5 ± 3.2%; n = 8; Fig. 5D), suggesting that cochlin might be inhibiting the channel function through a C-type gate mechanism. In agreement with these results, a diminished sensitivity to shear stress was also observed in high K^+^ solution (+10.4 ± 3.9%; n = 8; Fig. 5D). Our electrophysiological experiments suggest a direct interaction of cochlin with TREK-1 channel but whether inhibitory/excitatory effects on channel current are directly related to cell shape remodeling it still is not known. In fact, TREK-1 effects on cell shape have been reported to be independent of its ion transport capability^11^.

**Figure 6:**
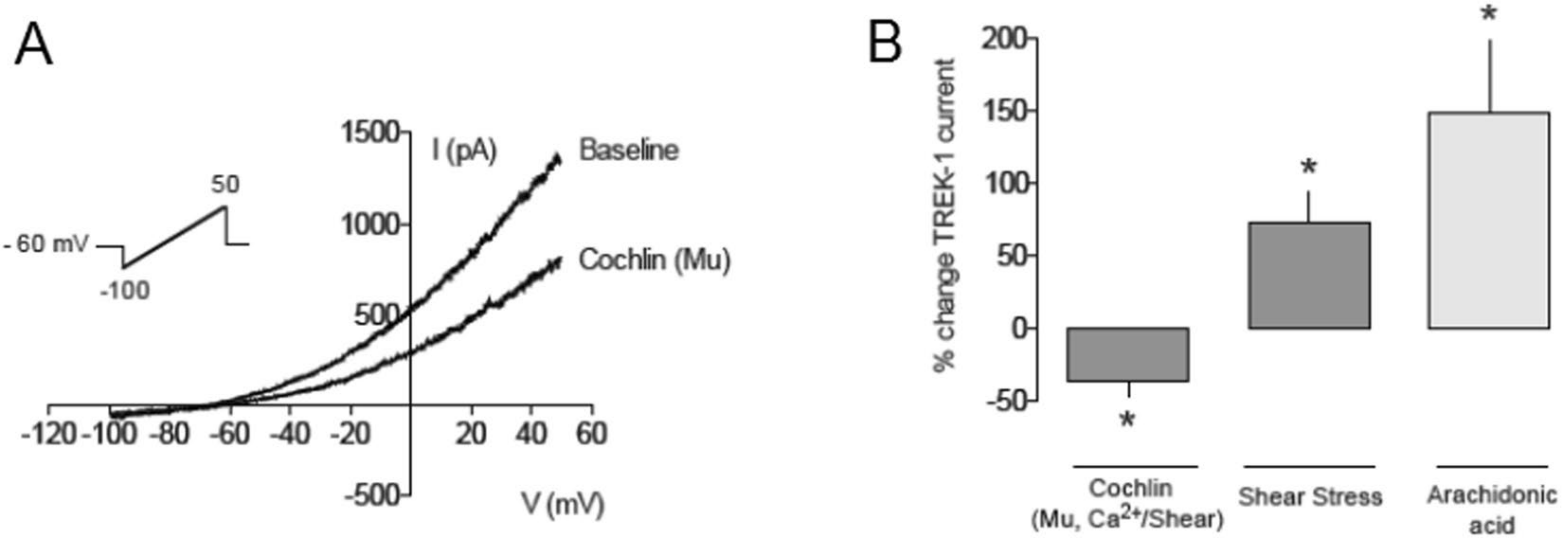
Whole-cell patch clamp recordings of TREK-1 in TM cells (**A**) TREK-1 current elicited by a voltage ramp from −100 to +50 mV in basal conditions and after addition of multimerized cochlin (10 nM) applied to the bath in a human trabecular meshwork cell (HTM-5) expressing hTREK-1. (**B**) Quantification of the effects of cochlin on hTREK-1 current in transiently transfected HTM-5 cells: multimerized cochlin (Mu, 10 nM; n = 5); shear stress (n = 5) and arachidonic acid (n = 5; 20 µM). *p < 0.05, Student’s t-test vs. basal current.

To assess whether the effects of cochlin on TREK-1 current were similar to these found in HEK293 cells, we used a trabecular meshwork cell line derived from a normotensive patient^25^ transiently transfected with TREK-1. TREK-1 current was recorded with a voltage ramp from −100 to +50 mV and then challenged with multimeric cochlin as previously described. Multimeric cochlin induced a statistically significant decrease in TREK-1 current of 36.8 ± 11.2%; n = 5; p < 0.05 vs. baseline; Fig. 6A,B). The effect of cochlin was opposite to the well-known effects of shear stress stimulation by fluid flow or arachidonic acid (20 µM), which significantly increased TREK-1 current by 72.1 ± 21.9% and 148.7 ± 50.1%, respectively (n = 5 each; Fig. 6B).

## Discussion

The mechanosensing performed by cochlin in the solution phase in concert with mechanotransduction by TREK-1 on the cell surface is a novel finding. We demonstrate here that their interaction leads to previously shown cytoskeletal remodeling in the TM. Impairment of aqueous humor outflow is linked to spatial changes in the ECM and potential remodeling of the cytoskeleton^26, 27^. In other systems, TREK-1 is recognized as working independently in order to perform its mechanotransducing functions yet in the trabecular meshwork, cochlin acts as a mechanosensing molecule assisting TREK-1 mechanotransduction. The presence of cochlin helps in facilitating TREK-1’s mechanotransducing properties in a low fluid flow regime compared to the high fluid flow regime of the kidneys. Interestingly, studies performed in alveolar epithelial cells have demonstrated that the location or regime of TREK-1 expression causes differences in function. In this cell type, TREK-1 deficiency correlates with decreasing actin stress fibers and TREK-1 overexpression correlates with increasing stress fibers^28^. Our results allude to a difference in effect on TM cells in the presence of multimeric cochlin possibly due to cell type as well as location.

The importance of cochlin in IOP regulation has previously been demonstrated through silencing of cochlin using shRNA, which resulted in decreased IOP^7, 18^. TREK-1 downregulation may significantly reduce the sensitivity of cells to detect mechanical stimulus necessary for collagen expansion (Fig. 2C) and is consistent with the observed decreased fluorescein transport (Fig. 2D). It is noted that the fluorescein dye used in these experiments is transported via transcellular transport in some cell types^29^. It is important to point out that fluorescein experiments were performed using only monomeric cochlin which explains the potential lack of consistency with physiological experiments as described below. The shRNA used for these experiments penetrates all three cell layers as seen from analysis of each cell layer separately. These findings also further support the necessary presence of both TREK-1 and cochlin in order to elicit a change in the cellular architecture as previously shown for these two molecules separately^7, 18^. The TREK-1 and cochlin interaction is not sufficiently supported with the collagen gel assays and fluorescein transport assays (Fig. 2C and D) alone but they lay the foundation for the remainder of our studies. Taken together with our functional experiments as discussed below and the remainder of our experiments, the role that these two molecules are crucial in aqueous humor outflow regulation is supported. It is important to highlight the fact that in the eye, due to the particular location of TM tissue and its architecture, remodeling will modulate aqueous humor outflow and may affect IOP homeostasis^18^. The change in cell shape as well as the actin cytoskeleton staining in the presence of multimeric cochlin is consistent with our observations previously described in TM cells after cochlin exposure^7, 18^. It is evident that the multimeric cochlin elicits a response from cells that causes them to become more elongated and spindle like in conformation (Fig. 3). Also, the presence of TREK-1 in the vicinity of cochlin together with demonstration of interaction *in vitro* and *in vivo* suggests the functional influence these elements have upon each other. Fluorescence resonance energy transfer (FRET) was attempted for cochlin and TREK-1 but did not produce sufficient data as technical difficulties were experienced as a result of the various domains present in TREK-1. Both molecules proved to be too large to exhibit any consistent resonance data.

Previously, we postulated a model in which multimerized cochlin binding to TREK-1 changes cell shape and motility. These experiments provide further evidence to support this model. TREK-1 mediated cytoskeletal rearrangement appears to be independent of TREK-1 channel activity^11^. TREK-1 activation may allow the cell to “relax” and increase outflow in the normal state similar to the effect produced by the high-conductance calcium dependent K^+^ channel (BKCa)^30, 31^. In fact, monomeric cochlin produced a small but significant increase in TREK-1 current, thus potentially favoring cell relaxation (Fig. 5D). This data supports the idea that monomeric cochlin interacts with TREK-1 in the physiological environment causing a small increase in TREK-1 current resulting in a positive effect on outflow and a reduction in IOP. In the diseased model, as supported by our data, multimerized cochlin decreases TREK-1 current, which may in turn decrease outflow, as a result of the cellular structure changes induced by the interaction of both proteins. The negative effects of cochlin are elicited when it interacts with TREK-1 in its multimerized form causing an inhibition in TREK-1 current and cellular architecture rearrangement that may contribute to impedance in outflow followed by an increase in IOP. It is important to note that in other regimes, such as the uterus during pregnancy, TREK-1 expression is seen to decline in order to promote a contractile state^32^. These findings may share functional characteristics with TREK-1 in the TM in the presence of multimeric cochlin. Our data, for the first time, suggests the involvement of an interaction of cochlin and TREK-1 in glaucoma and renders this interaction a target for therapy. Further studies will elucidate as to how TREK-1 or cochlin separately or together can be manipulated as potential therapeutic targets for flow associated disease pathologies.

## Methods

The study protocols were approved by the University of Miami IACUC. The methods were carried out in accordance with the approved guidelines.

### Cochlin transgene and cochlin and TREK-1 shRNA lentivirus production

To overexpress cochlin in the TM of congenic DBA/2J-Gpnmb+/SjJ mice, a cochlin transgene bearing lentivirus was constructed in HEK293T cells (cat# 293T/17 (CRL-11268), ATCC, Manassas, VA). The cochlin expression clone (cat# EX-Q0226-Lv31, GeneCopoeia Inc., Rockville, MD) was packaged into a lentiviral vector using the Lenti-Pac FIV expression packaging kit and the protocol provided by the manufacturer. This protocol typically yielded 10^7^ infectious units/mL of the recombinant lentivirus. The cochlin gene (COCH) used to produce the cochlin expression vector was human (NM_004086).

To down-regulate TREK-1 expression in the TM of DBA/2J-Gpnmb+/SjJ mice, TREK-1 shRNA virus was made in HEK293T cells using the Trans-Lentiviral™ GIPZ Packaging System (cat# TLP4614, Open Biosystem, Huntsville, AL) and the protocol provided by the manufacturer. This typically yielded a viral stock of 108 transduction units (TU)/mL. The shRNA used was a set of 5 clones. Transfection efficiency was determined to be 70%. This efficiency was validated by resolving equal amounts of protein by SDS-PAGE and detecting TREK-1 expression via western blot. The membrane was stripped and re-probed for GAPDH in order to confirm equal loading.

The mice that were given an intracameral injection with the cochlin over-expression vector alone or TREK-1 down-regulation vector with cochlin over-expression vector were measured for intraocular pressure before injection. The mice were anaesthetized with an intraperitoneal injection (0.1 μL) of ketamine (100 mg/kg) and xylazine (9 mg/kg) prior to IOP measurement. The IOP was taken using a hand held tonometer, TonoLab (Colonial Medical Supply, Franconia, NH) after the mouse of interest failed to respond to touch. The IOP was measured throughout the course of the study following this procedure.

### Fluorescein dye transport and gel expansion assay

An Ussing-type chamber (cat# USS1L, World Precision Instruments Inc. (WPI), Sarasota, FL) was used to measure fluorescein flow (fluorescein dye) across a polyvinylidene fluoride (PVDF) membrane (cat# 75696E, Pall Life Sciences, Pensacola, FL). TM cells were cultured on the PVDF membrane. Before plating the cells, a layer of collagen matrix (Rat Tail Collagen, cat# 354249, BD Biosciences, San Jose, CA) was formed on the membrane to facilitate cell adherence. The cells were allowed to form a confluent monolayer over a period of 16–24 h, following the addition of another cell layer. This process was repeated to ultimately achieve a confluent tri-layer of cells. The cells were transfected with DNA of interest (TREK-1 + cochlin, TREK-1 + RPE65, TREK-1 only, transfection agent (Lipofectamine 2000, Invitrogen Inc., Carlsbad, CA), or non-transfected control). Twenty-four to thirty-six hours post-transfection the membranes were placed between the hemi-chambers of the apparatus connected to a single-channel peristaltic pump (cat# 151922, Watson-Marlow, Wilmington, MA) with 1X PBS as the bathing medium. To measure the flow across the membrane, sodium fluorescein dye was used (1:100 dilution of 1 mg/mL). A set volume of the dye was introduced into one hemi-chamber. After 5 minutes, equal volume was aspirated out from the opposite side of the membrane through the opposing hemi-chamber opening. The fluorescein concentration was calculated using a spectrophotometer.

The collagen gel assay was performed following a published procedure^33^. A hydrogel solution was prepared in a serum-free environment with the following: 10X MEM (cat# 11430, Invitrogen), sodium bicarbonate (cat# S5761, Sigma-Aldrich), L-glutamine (cat# G6392, Sigma-Aldrich) and HEPES buffer (cat# 15630, Invitrogen). The following solution was aliquoted into separate Eppendorf tubes for each transfection type. Transfection complexes were prepared in separate tubes containing a mixture of the transfection agent (Lipofectamine 2000, Invitrogen) and desired DNA vectors with a transfection agent ration (w/v) of 0.4 μg/μL.

Trabecular meshwork cells were obtained from a >90% confluent layer of trypsin-treated 25 cm^2^ cell culture flask (cat# 353109, Becton Dickinson, Franklin Lakes, NJ) centrifuged for 5 minutes at 800 rpm (77xG) cell pellet was then re-suspended in serum free DMEM 1X culture media (cat# 15-013-CV, CellGro, Corning), thoroughly mixed and equally aliquoted into each respective transfection-complex containing vessel. The reaction was allowed to incubate for 45 minutes, after which it was terminated through addition of cell culture media (DMEM1X + 10% FBS) followed by the addition of the previously prepared hydrogel solution and the rat tail collagen (BD Biosciences) to initiate gel polymerization. The suspension was gently mixed and aspirated into 1 mL single use needle syringe (Henk-Sass-Wolf). This suspension was injected into a borosilicate glass capillary tube with an inner diameter of 0.75 mm (cat# TW100-6, WPI) to approximately half of its volume. A digital picture snapshot was taken of all capillaries against blank background with a millimeter scale following the hydrogel injection inside a custom-built fixed height transparent plexiglass chamber and was repeated every 24 h for 48 h. The hydrogel containing capillaries were incubated inside a specially prepared moisture chamber to prevent dehydration as well as gel displacement and were kept inside a cell culture incubator at 37 °C and 5% CO_2_. Photographs obtained using a digital camera was analyzed using NIH ImageJ (v.1.43 u) software. Lengths were measured between the opposite ends of the gel and subjected to statistical analysis using Microsoft Excel 2007 (Microsoft Corp., Redmond, WA).

### Western blot analysis of cochlin

Cochlin was purified using established procedures^34^. Approximately 10 ug of the purified exogenous cochlin was subjected to shear stress after purification. To produce shear stress, the purified cochlin was passed through a 30-gauge needle 10–20 times. The preparation was then subject to western blot analysis for cochlin detection and TREK-1 detection. These analysis were performed under non-reducing but denaturing conditions as wells as reducing and denaturing conditions. For Western blot the proteins were separated on 4–20% Tris-glycine gel (cat# EC6028BOX, Invitrogen, Carlsbad, CA) and then transferred to PVDF membranes (cat# 162-0219, BioRad Laboratories, Hercules, CA). For cochlin identification, custom antibodies against cochlin peptides (KR LKK TPE KKT GNK DC from cochlin coding region 147–162 designated as hCochlin# 1; ZCZ TYD QRT EFS FTD YST KEN; from cochlin coding region 412–429 designated as hCochin#2; and CZ DDL KDM ASK PKE SH from cochlin coding region 358–371 designated as hCochlin#3, Aves labs Inc., Tigard, OR) were used at ~5 μg/mL^34^. For TREK-1 identification, a primary antibody from Abcam was utilized at ~5 μg/mL (TREK-1: cat# ab83932, Abcam, Cambridge, MA). A secondary antibody conjugated to horseradish peroxidase (goat anti-chicken cat# H-1004, Aves Labs Inc.; goat anti-rabbit cat# ab6721, Abcam, Cambridge, MA) was added and cochlin/TREK-1 proteins were detected using enhanced chemiluminescent substrate (ECL) (cat# 32106, Pierce Thermo Fisher Scientific Inc, Rockford, IL). GAPDH (anti-GAPDH cat# ab22556, Abcam, Cambridge, MA) was used as a loading control.

### Reciprocal immunoprecipitation

Human TM was dissected from enucleated eyes obtained from the Bascom Palmer Eye Bank (BPEI). These eyes were taken from normal donors between 40–85 years of age. All human TM cells used were authenticated by detection of myocilin with repeated dexamethasone treatments before experimental use. TM was dissected from the enucleated eyes, finely minced, and protein extraction was performed using 50 mM Tris-HCl, ph 7.5, 125 mM NaCl, and 0.1% genapol (cat# 345794, EMD Biosciences, La Jolla, CA). To produce shear stress, cochlin was passed through a 30-gauge needle 10–20 times. The 100 µg of protein extract was added with 1–2 µg of cochlin (either native or shear stressed) in an Eppendorf tube.

After incubating the above tubes at room temperature for 2 h, 50 µL of TREK-1 antibody coupled to magnetic beads (cat# 88802, ThermoScientific Pierce Protein A/G magnetic beads) was added and again incubated for 2 h at room temperature. Magnetic beads were removed using a magnet. The resultant precipitate was re-suspended in 200 µL of 1X PBS and magnetic beads were collected. The supernatant was discarded and was followed by the addition of 50 µL of 100 mM glycine (pH 3.0) to the magnetic beads. The supernatant was collected and neutralized with 1.5 M Tris-HCl buffer, pH 8.8 (cat# 161-0798, Bio-Rad Laboratories). Proteins were subjected to Western blot analysis and probed for cochlin (usually hCochlin#3, Aves Labs Inc.).

The tubes were incubated with magnetic beads (cat# 88802, ThermoScientific Pierce Protein A/G magnetic beads) coupled to chicken anti-cochlin antibody (hCochlin#3, Aves Labs Inc.). The magnetic beads were collected and washed in 200 µL of 1X PBS. The supernatant was discarded and 50 µL of 100 mM glycine (pH 3.0) was added to the magnetic beads. The supernatant was collected and neutralized with 1.5 M Tris-HCl buffer, pH 8.8 (cat# 161-0798, Bio-Rad Laboratories). The proteins were subjected to Western blot analysis and probed with antibodies against TREK-1 (cat# ab83932, TREK-1, Abcam). Reciprocal immunoprecipitation for cochlin (hCochlin#3, Aves Labs Inc.) was carried out following a similar protocol as described above.

### Immunocytochemistry on cochlin treated TM cells

Human normal TM cells (NTM) were cultured in 12 well plates (cat# 3513, Costar, Corning Incorporated) on circular microscope cover slides (cat# 48380-068, VWR International) with serum-free cell culture media (cell culture media 1X DMEM (cat# 15-013-CV, CellGro, Corning). Cells were incubated in an incubator at 37 °C and 5% CO_2_ for 24 h. At the 24 h mark, monomeric cochlin or multimeric cochlin was added to specified wells at a concentration of 10 ug per well. Multimeric cochlin was produced by passing the monomeric cochlin through a 30-gauge syringe 15–20 times before adding to the well. Following incubation, media was removed and cells were washed 3X with 1X PBS (cat# 21-040-CV, Mediatech Inc., Manassas, VA) then fixed with 1% paraformaldehyde (cat# 15710, Electron Microscopy Sciences, Hatfield, PA) for 15 minutes. After fixation cells were washed 3X with 1X PBS before blocking. Cells were blocked with 1X PBS + 0.2% bovine serum albumin (BSA) (cat# 2910, Fraction V, EMD Chemicals, Gibbstown, NJ) for 30 minutes. The primary antibody was added for cochlin in 1:200 dilution (cochlin: hCochlin#3 Aves Labs Inc.). After incubating overnight at 4 °C, primary antibody was washed out with 1X PBS + 0.2% BSA three times for 10 minutes per wash. The corresponding secondary antibody was added in 1:1000 dilution (Donkey anti-chicken FITC, cat#ab63507, Abcam) and incubated for 1 h at room temperature. Following secondary antibody incubation, slides were washed three times for 10 minutes per wash with 1X PBS + 0.2% BSA. Slides were then incubated with 100 nM rhodamine phalloidin (cat# PHDR1, Cytoskeleton) for 30 minutes to stain the actin cytoskeleton. Slides were then washed three times for 10 minutes per wash with 1XPBS. The cover slides were then mounted on glass microscope slides (cat# 48300-0205, VWR International, West Chester, PA) and stained with DAPI Vectashield (cat# H-1200, Vector Laboratories). Prepared slides were imaged using Leica DM 6000 B confocal microscope (Leica, Inc.). Intensity of fluorescence was measured in a relative arbitrary unit under the same settings and conditions for each sample using ImageJ software. Cellular conformation was confirmed via individual counting performed by five different individuals blinded to cell treatment. Averages were taken from these calculations and presented as an arbitrary measurement of cells/area.

### Immunocytochemistry on arachidonic acid (AA) treated TM cells

Human normal TM cells (NTM) were cultured in 12 well plates (cat# 3513, Costar, Corning Incorporated) on circular microscope cover slides (cat# 48380-068, VWR International) with serum-free cell culture media (cell culture media 1X DMEM (cat# 15-013-CV, CellGro, Corning). Cells were incubated in an incubator at 37 °C and 5% CO_2_ for 24 h. At the 24 h mark, specific wells of cells were treated with 20 µM AA diluted in serum-free DMEM media. Along with the AA, 5 µM of indomethacin, a cyclooxigenase inhibitor, was added in order to inhibit AA metabolism for 30 minutes. Following treatment, cells were washed 3X with 1X PBS (cat# 21-040-CV, Mediatech Inc., Manassas, VA) then fixed with 1% paraformaldehyde (cat# 15710, Electron Microscopy Sciences, Hatfield, PA) for 15 minutes. After fixation cells were washed 3X with 1X PBS before blocking. Cells were blocked with 1X PBS + 0.2% bovine serum albumin (BSA) (cat# 2910, Fraction V, EMD Chemicals, Gibbstown, NJ) for 30 minutes. The primary antibody was added for TREK-1 in 1:200 dilution (cat#: sc-398449, Santa Cruz Biotechnology, Inc.). After incubating overnight at 4 °C, primary antibody was washed out with 1X PBS + 0.2% BSA three times for 10 minutes per wash. The corresponding secondary antibody was added in 1:1000 dilution (Goat anti-mouse Alexa Fluor 488, cat#: 948490, Invitrogen Molecular Probes) and incubated for 1 h at room temperature. Following secondary antibody incubation, slides were washed three times for 10 minutes per wash with 1X PBS + 0.2% BSA. Slides were then incubated with 100 nM rhodamine phalloidin (cat# PHDR1, Cytoskeleton) for 30 minutes to stain the actin cytoskeleton. Slides were then washed three times for 10 minutes per wash with 1XPBS. The cover slides were then mounted on glass microscope slides (cat# 48300-0205, VWR International, West Chester, PA) and stained with DAPI Vectashield (cat# H-1200, Vector Laboratories). Prepared slides were imaged using Leica DM 6000 B confocal microscope (Leica, Inc.). Intensity of fluorescence was measured in a relative arbitrary unit under the same settings and conditions for each sample using ImageJ software.

### Immunohistochemistry on human TM sections

Human TM sections embedded in paraffin were deparaffinized, hydrated for 20 min with 1X phosphate-buffered saline (PBS) (cat# 21-040-CV, Mediatech Inc., Manassas, VA), and blocked in 1X PBS + 0.2% bovine serum albumin (BSA) (Fraction V, cat# 2910, EMD Chemicals, Gibbstown, NJ) for 30 m. The primary antibody was added for cochlin and TREK-1 in 1:200 dilution (cochlin: hCochlin #3 (cat# 5007/5008) Aves Labs Inc., TREK-1: cat# ab83932, Abcam, Cambridge, MA). After incubating overnight at 4 °C, primary antibody was washed out with 1X PBS + 0.2% BSA 3 times for 10 m per wash. The corresponding secondary antibody was added in 1:1000 dilution (Alexa594, cat# A11042, Invitrogen; Alexa488: cat# ab150073, Abcam) and incubated for 1 h at room temperature. The sections were then mounted on glass microscope slides (cat# 48300-0205, VWR International, West Chester, PA) and stained with DAPI Vectashield (cat# H-1200, Vector Laboratories). Thus prepared slides were imaged using Leica DM 6000 B confocal microscope (Leica, Inc.).

### Whole cell patch clamp recording

HEK293T and TM cells, cultured at 37 °C and 5% CO_2_ in DMEM with 10% FBS, 1% penicillin/streptomycin and 1% glutamine, were seeded in 12-mm dishes 24 h post transfection. Human trabecular meshwork cell line, derived from an 18-year-old non-glaucomatous male donor, was kindly provided by Alcon Laboratories (Fort Worth, TX)^25^. Cells were transiently transfected with pIRES_2_-EGFP vector alone (control) or with pEZ-Lv205-hTREK-1 (GeneCopoeia, Inc.) using FuGene transfection reagent (Roche), according to the manufacturer’s instructions. Cells were used for patch-clamp experiments 24–48 h after transfection. For the TM cells, the K2 Transfection System from Biontex Laboratories GmbH (Planegg/Martinsried, Germany) was used following manufacturer instructions.

Electrophysiological recordings were performed with a patch-clamp amplifier (Axopatch 200B, Molecular Devices, Union City, CA). Patch electrodes were fabricated in a Flaming/Brown micropipette puller P-97 (Sutter instruments). Electrodes had a resistance between 2–4 MΩ when filled with intracellular solution (in mM): 135 KCl, 2.1 CaCl_2_, 2.5 MgCl_2_, 5 EGTA, 2.5 ATP, 10 HEPES at pH 7.3. Bath solution was (in mM): 145 NaCl, 5 KCl, 2 CaCl_2_, 2 MgCl_2_, 10 HEPES at pH 7.4. The osmolality of the isotonic solution was adjusted with sorbitol to ≈300 mOsm/Kg. For C-type gate experiments in high K^+^ solution, the solution used (in mM): 15 NaCl, 135 KCl, 2 CaCl_2_, 2 MgCl_2_, 10 HEPES at pH 7.4.

Membrane currents were recorded in the whole-cell patch clamp configuration, filtered at 2 kHz, digitized at 10 kHz and acquired with pClamp 10 software. Data was analyzed with Clampfit 10 (Molecular Devices) and Prism 4 (GraphPad Software, Inc., La Jolla, CA). Series resistance was always kept below 15 MΩ and compensated at 70–80%. All recordings were done at room temperature (22–23 °C). Shear stress stimulation in transfected cell lines was achieved by increasing bath perfusion rate from 0 or 0.1 mL/min (baseline) to 1 mL/min (shear stress). In the whole cell configuration, cells were held at −60 mV and 1 s voltage ramps from −100 to +50 mV were used to record TREK-1 mediated current. For patch clamp experiments, monomeric cochlin (Mo) was used diluted in recording solution in the absence of Ca^2+^ without any prior preparation. Multimeric cochlin was produced by passing the monomeric cochlin through a 30-gauge syringe 15–20 times before adding to the well (Mu, shear) or by adding Ca^2+^ to the solution where cochlin was prepared (Mu, Ca^2+^). Arachidonic acid was added to the bath at a final concentration of 20 µM.
