# Erratum: Interaction of cochlin and mechanosensitive channel TREK-1 in trabecular meshwork cells influences the regulation of intraocular pressure

**DOI:** 10.1038/s41598-017-04858-4

**Published:** 2017-07-20

**Authors:** Teresia A. Carreon, Aida Castellanos, Xavier Gasull, Sanjoy K. Bhattacharya

**Affiliations:** 1 Bascom Palmer Eye Institute, University of, Miami, Miami Florida USA; 2 Department of Biochemistry and Molecular Biology, University of Miami, Miami, USA; 3 Department of Biomedicine, University of Barcelona, Barcelona, Spain; 4 Institut d’Investigaciones Biomediques August Pi I Sunyer (IDIBAPS), Barcelona, Spain; 5 Institute of Neurosciences, University of Barcelona, Barcelona, Spain

*Scientific Reports* **7**:452; doi:10.1038/s41598-017-00430-2; Article published online 28 March 2017

The original version of this Article contained an error in the Abstract, which now reads:

“In the eye, intraocular pressure (IOP) is tightly regulated and its persistent increase leads to ocular hypertension and glaucoma. We have previously shown that trabecular meshwork (TM) cells might detect aqueous humor fluid shear stress via interaction of the extracellular matrix (ECM) protein cochlin with the cell surface bound and stretch-activated channel TREK-1. We provide evidence here that interaction between both proteins are involved in IOP regulation. Silencing of TREK-1 in mice prevents the previously demonstrated cochlin-overexpression mediated increase in IOP. Biochemical and electrophysiological experiments demonstrate that high shear stress-induced multimeric cochlin produces a qualitatively different interaction with TREK-1 compared to monomeric cochlin. Physiological concentrations of multimeric but not monomeric cochlin reduce TREK-1 current. Results presented here indicate that the interaction of TREK-1 and cochlin play an important role for maintaining IOP homeostasis.”

This has now been corrected in the HTML and PDF versions of this Article.

